# Assessing the Sensitivity of Different Life Stages for Sexual Disruption in Roach (*Rutilus rutilus*) Exposed to Effluents from Wastewater Treatment Works

**DOI:** 10.1289/ehp.7921

**Published:** 2005-06-14

**Authors:** Katherine E. Liney, Susan Jobling, Jan A. Shears, Peter Simpson, Charles R. Tyler

**Affiliations:** 1 Environmental and Molecular Fish Biology Group, School of Biosciences, Hatherly Laboratories, Exeter, United Kingdom; 2 Aquatic Ecotoxicology Research Group, Department of Biological Sciences, Brunel University, Middlesex, United Kingdom; 3 Environment Agency, Waterlooville, United Kingdom

**Keywords:** differentiation, effluent, endocrine, fish, wastewater treatment works

## Abstract

Surveys of U.K. rivers have shown a high incidence of sexual disruption in populations of wild roach (*Rutilus rutilus*) living downstream from wastewater treatment works (WwTW), and the degree of intersex (gonads containing both male and female structural characteristics) has been correlated with the concentration of effluent in those rivers. In this study, we investigated feminized responses to two estrogenic WwTWs in roach exposed for periods during life stages of germ cell division (early life and the postspawning period). Roach were exposed as embryos from fertilization up to 300 days posthatch (dph; to include the period of gonadal sex differentiation) or as postspawning adult males, and including fish that had received previous estrogen exposure, for either 60 or 120 days when the annual event of germ cell proliferation occurs. Both effluents induced vitellogenin synthesis in both life stages studied, and the magnitude of the vitellogenic responses paralleled the effluent content of steroid estrogens. Feminization of the reproductive ducts occurred in male fish in a concentration-dependent manner when the exposure occurred during early life, but we found no effects on the reproductive ducts in adult males. Depuration studies (maintenance of fish in clean water after exposure to WwTW effluent) confirmed that the feminization of the reproductive duct was permanent. We found no evidence of ovotestis development in fish that had no previous estrogen exposure for any of the treatments. In wild adult roach that had previously received exposure to estrogen and were intersex, the degree of intersex increased during the study period, but this was not related to the immediate effluent exposure, suggesting a previously determined programming of ovotestis formation.

A widespread occurrence of sexual disruption has been reported in freshwater and marine fish in Europe (Hecker et al. 2002; Jobling et al. 1998; Lye et al. 1997; Minier et al. 2000; Vethaak et al. 2002), Japan (Hashimoto et al. 2000), and the United States (Folmar et al. 2001; Munkittrick et al. 1998), and some of these effects have been linked with exposure to endocrine-disrupting chemicals emanating from wastewater treatment works (WwTW) effluents. Sexual disruption is defined as disruption of gonadal development in terms of either altered duct formation and/or development of ovotestis. The most commonly documented feminizing effect of WwTW effluents in fish is induction of vitellogenin (VTG; an estrogen-dependent yolk precursor) (Purdom et al. 1994). Other feminizing effects of WwTW effluents include altered sex steroid hormone levels in adult and juvenile fish (Folmar et al. 1996; Hecker et al. 2002), impaired gonadal development in adults and juveniles (Hemming et al. 2001; Jobling et al. 2002a), altered timing of sexual differentiation in early life stages (Rodgers-Gray et al. 2001), and gonadal intersex (gonads containing both male and female characteristics) (Jobling et al. 1998). Gonadal intersex includes disruption of the gonadal duct, where the male duct is feminized to form a female-like ovarian cavity, and/or the presence of both male and female germ cells within the same gonad (Nolan et al. 2001). Intersex has been reported in a wide variety of gonochoristic species of wild freshwater and marine fish worldwide (Allen et al. 1999; Harshbarger et al. 2000; Hashimoto et al. 2000; Jobling et al. 1998; Kavanagh et al. 2004; van Aerle et al. 2001).

The most extensive studies on the feminizing effects of WwTW effluents in wild fish have been conducted on the roach (*Rutilus rutilus*) living in U.K. rivers where surveys of > 50 river sites have found a high incidence of intersex in roach populations living downstream from WwTW effluent outlets (Jobling et al. 1998). The incidence and severity of the intersex condition were correlated with the proportion of WwTW effluent in the river and the population equivalent of the WwTWs (strength of the effluent) (Jobling et al. 1998). Recently, the predicted amount of steroid estrogen discharged from WwTWs was shown to correlate with the degree of feminization in wild intersex roach living immediately downstream of the WwTW discharge (Jobling et al. in press). Importantly, intersex roach have been shown to have both reduced milt volume and sperm density (Jobling et al. 2002a) and reduced fertility (Jobling et al. 2002b); thus, there is the potential for the intersex condition to lead to population-level effects.

Laboratory studies have shown that it is possible to induce gonadal duct disruption and germ cell disruption in early-life-stage (ELS) fish by exposing them to some of the estrogenic chemicals found in WwTW effluent, but generally this occurs only at concentrations higher than those found in the environment (Gimeno et al. 1997, 1998; Gray et al. 1999; Knorr and Braunbeck 2002; Koger et al. 2000; Yokota et al. 2000). Our previous work has shown that exposure of roach to an estrogenic WwTW effluent during early life [50–150 days posthatch (dph)] induced VTG synthesis and gonadal duct disruption (feminization) but not the simultaneous presence of male and female germ cells in the same gonad (Rodgers-Gray et al. 2001). The precise timing of the genetic programming for sex (germ cell) differentiation in the roach, however, is not known, and it may be initiated before 50 dph, and thus the relevant window for sexual disruption may not have been captured in that study.

Less is known about the potential plasticity of differentiated germ cells and thus the sensitivity of these cells for estrogenic disruption in gonochoristic fish. Juvenile medaka (that had undergone sexual differentiation) have been shown to undergo sex reversal when exposed to steroids, both male to female and female to male (Yamamoto 1953, 1958). Induction of intersex in sexually mature male medaka has also been reported after treatment with estradiol (Egami 1955a, 1955b; Kang et al. 2002; Shibata and Hamaguchi 1988). The conclusions drawn from these studies were that testis-ova were formed during the process of spermatogenesis and arose as a consequence of the transformation of spermatogonia B into female sex cells (oocytes) (Shibata and Hamaguchi 1988). In roach during the post-spawning period, there is a short interval of gonadal regression before further germ cell proliferation and renewed gonadal growth. It is possible that this window of germ cell proliferation in adult roach offers a window of enhanced sensitivity for germ cell disruption, when circulating endogenous steroid hormones are at low concentrations (Vainikka et al. 2004).

Estrogenic chemicals commonly detected in WwTW effluent and receiving waters include natural steroid estrogens, synthetic estrogens, alkylphenolic compounds, phthalates, bisphenols, and various pesticides and herbicides (Azevedo et al. 2001; Desbrow et al. 1998; Zhang et al. 2004). Steroid estrogens and some alkylphenolic chemicals are often found in WwTW effluents at concentrations sufficient to induce vitellogenic responses in laboratory exposures (Routledge et al. 1998; Tyler and Routledge 1998). Natural steroid estrogens (Kang et al. 2002; Koger et al. 2000), the synthetic steroid estrogen ethinylestradiol (Metcalfe et al. 2001), and alkylphenolic compounds (Gray and Metcalfe 1997; Gronen et al. 1999) have also been shown to induce intersex in fish, but generally at concentrations higher than those present in WwTW effluent discharges. Steroidal estrogens and alkylphenolic chemicals (and other environmental estrogens) have also been shown to be interactive (additive) in their effects (Silva et al. 2002; Thorpe et al. 2001, 2003). Together, these data strongly support the hypothesis that steroidal estrogens and alkylphenolic chemicals are key compounds in the development of feminized responses, including intersex in wild roach populations living in U.K. rivers.

Feminized responses in roach exposed to two estrogenic WwTWs were investigated during life stages of potential sensitivity for intersex induction (periods encompassing germ cell division). The developmental stages studied encompassed the period of sexual differentiation, from fertilization through the completion of the period of gonadogenesis and the postspawning period in adult fish. Given the likely role of steroidal estrogens and alkylphenolic chemicals in the induction of intersex in wild roach, these chemicals were measured in the effluents for each of the exposure studies conducted.

## Materials and Methods

### Experimental design.

We set up experiments to investigate the effects of treated wastewater effluent on sexual development in roach during two life history stages of perceived sensitivity for the disruption of sexual development, the first encompassing the period of sexual differentiation in early life and the second in adult fish during germ cell proliferation after the annual spawning event.

### Treated wastewater effluents.

Roach were exposed to effluents from two U.K. WwTWs (WwTW A and WwTW B) with different population equivalents and treatment processes. At WwTW A, industrial influent to the works included approximately 6% of the total influent, and the population equivalent of the works influent was approximately 137,000. WwTW B had a higher industrial component in the influent (24%), and the population equivalent was approximately 312,700, double that at WwTW A. Influents at both works received secondary treatment. At WwTW A, secondary treatment consisted of trickling filters and bubble-diffused air-activated sludge treatment. Secondary treatment at the WwTW B plant consisted of bubble-diffused air-activated sludge (60% of the flow) and biological phosphorus-removal–activated sludge (40% of the flow), which were then combined before discharge into the receiving rivers. Extensive studies on the effluent from WwTW A have shown it to be estrogenic to fish (Harries et al. 1999; Rodgers-Gray et al. 2001). The effluent from WwTW A is pumped into an estuary where roach do not live, and hence the possible effects of this effluent on sexual development and function in wild roach have not been investigated. Direct studies on the estrogenicity of the effluent from WwTW B had not been previously undertaken, but there is a high incidence of intersex in wild roach living in the river below the discharge of this effluent (Jobling et al. 1998).

### Feminized responses in roach exposed to WwTW effluent during early life (fertilization to 300 dph).

**Figure 1:**
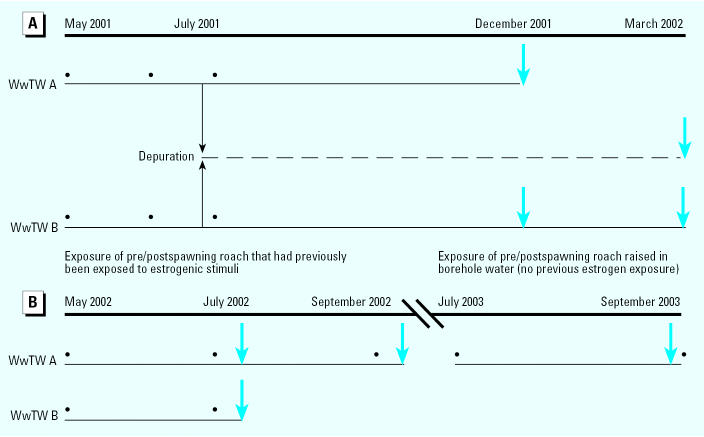
Experimental design for the effluent exposure studies. Bullets indicate sampling points for analytical chemistry (collections of 7-day composite samples of effluent). Blue arrows indicate sampling points for biological analyses. (*A*) ELS study in which fertilized roach eggs were deployed into graded concentrations of WwTW effluent in mesocosm systems at both sites and maintained until 200 dph (WwTW A) and 300 dph (WwTW B). At both sites at 60 dph (July 2001), 60 fish from each effluent concentration and from controls were transferred to clean water for depuration and were sampled at 300 dph. (*B*) Postspawning roach studies in which sexually maturing male roach that had previously received exposure to estrogen were exposed to effluent from WwTW A for 4 months and WwTW B for 2 months (adult exposure 2), and another group of males with no previous exposure to estrogen was exposed to effluent from WwTW B for 2 months (adult exposure 1).

Figure 1A shows the experimental design for investigation of the effects of the two WwTW effluents in roach during early life. In this study, fish were exposed to graded concentrations of effluent continually from fertilization until 300 dph to include the full period for germ cell differentiation in this species. Each exposure system consisted of six tanks supplied with graded concentrations of treated sewage effluents and diluent (river water for WwTW A and dechlorinated tap water for WwTW B). Nominal concentrations of effluent at WwTW A were 100, 40, 20, and 10%, with river water and dechlorinated tap water as controls. At WwTW B, nominal effluent concentrations were 100, 80, 40, 20, and 10%, with a control tank receiving dechlorinated tap water. The flow rate through each of the tanks was 5 L/min, and flow rate and water temperature were monitored daily. The tanks were aerated to ensure sufficient oxygen supply. Fertilized roach eggs were allowed to hatch in 50-L glass-reinforced plastic tanks held within the larger exposure tanks. From hatching, fry were fed newly hatched *Artemia* until approximately 60 dph, when commercial (estrogen-free) cyprinid food pellets were introduced (Calverton Fish Farm, Nottingham, UK). Fish were later released into the larger 600-L mesocosm tanks at approximately 70 dph. At 60 dph, 60 fish from each treatment were transferred to clean water to depurate in order to compare a short-term exposure to treated WwTW effluent in early life (fertilization to 60 dph) with a chronic exposure from fertilization through to the completion of sexual differentiation (300 dph). Biological sampling was carried out (where numbers of surviving fish were sufficient) at 200 dph and 300 dph.

### Feminized responses in adult postspawning roach exposed to WwTW effluents.

We carried out two experiments to investigate the effects of exposure to WwTW effluent on feminized responses, including germ cell development, during the postspawning period in adult male roach. These experiments were particularly focused on assessing whether exposure to WwTW effluents at this time was capable of inducing intersex (ovotestis). The study included roach that had no previous exposure to estrogen (naive fish) and had been hatched and reared to maturity in captivity and clean water (adult exposure 1), and wild roach that hatched and grew to maturity in the wild (and likely had some previous exposure to estrogen; adult exposure 2; Figure 1B).

#### Adult exposure 1 (WwTW A only).

One hundred twelve spermiating male roach (aged 3+) that had been reared throughout their lives in borehole water were obtained from the Calverton Fish Farm. Groups of 25 male fish were exposed to 100, 50, and 25% effluent (mixed with river water) or to river water alone at WwTW A for a 2-month period from July to September 2003. The flow rate through each of the tanks was 6 L/min, and this was monitored daily. The tanks were aerated to ensure sufficient oxygen supply. Fish were fed daily on commercial (estrogen-free) cyprinid pellets throughout the exposure. At the start of the study, 12 fish were sampled for biological analyses (preexposure sample), and the remaining fish were deployed into the tanks and subsequently sampled at the end of the study only.

#### Adult exposure 2.

In this study, spermiating wild roach were deployed into five tanks at WwTW A that were supplied with graded concentrations of effluent and river water. Nominal effluent concentrations were 100%, 50%, and 25%, with further tanks supplied with river water and dechlorinated tap water as controls. At WwTW B, four tanks were supplied with graded concentrations of effluent, and an additional tank received tap water only as a control. Nominal effluent concentrations were 100%, 50%, and 25%. Groups of 20 mature spermiating male roach of mixed age classes were exposed to each of the treatments at WwTWs A and B for periods of 4 months (WwTW A) and 2 months (WwTW B) beginning in July. The flow rate through each of the tanks at both WwTWs was 6 L/min, and this was monitored daily. The tanks were aerated to ensure sufficient oxygen supply. Fish were fed daily with commercial cyprinid pellets. At the start of the experiment, 20 fish were sampled for biological analyses (preexposure sample)

### Measurement of steroid estrogens, alkylphenolic chemicals, and bisphenol A in the effluents.

Full-strength WwTW effluent samples from both WwTWs were collected during the studies. Seven-day composite WwTW effluent samples were collected from both WwTWs (Figure 1) and analyzed for the steroid estrogens 17β-estradiol, estrone, and 17α-ethinylestradiol and for the alkylphenolic compounds octylphenol, nonylphenol, and nonylphenol mono- and diethoxylates. Bisphenol A was also measured. Daily samples (2.5 L) of full-strength WwTW effluent were collected and refrigerated until processing (within 24 hr). The methods used to measure these chemicals were described by Kelly (2000) and Rodgers-Gray et al. (2000). In brief, the estrogenic chemicals were immobilized on a C18 silica-bonded solid-phase extraction column, eluted, and analyzed by gas chromatography/mass spectrometry. Chemical analysis was carried out by the Centre for Environment, Fisheries and Aquaculture Science (Burnham on Crouch, UK).

### Biological sampling.

In the ELS roach study, at each sampling point fish from each treatment group were sacrificed with a lethal dose of anesthetic [MS-222, according to Home Office recommendations; Animals (Scientific Procedures) Act 1986]. For VTG analysis, 30 fish were placed into cryovials, frozen on dry ice, and then transferred for storage at −20°C. For gonadal histology, 30 fish were fixed *in toto* for 24 hr in Bouin’s solution and stored in 70% industrial methylated spirits (IMS) before processing for histology.

In the adult fish trials, individual fish were anesthetized [MS-222, according to Home Office recommendations; Animals (Scientific Procedures) Act 1986], and blood was collected via the caudal sinus with heparinized syringes. The fish were then sacrificed [Schedule 1 method, according to Home Office recommendations; Animals (Scientific Procedures) Act 1986]. Aprotinin (2 trypsin inhibitor units/mL) was added to each blood sample; the blood was then centrifuged at 15,000 rpm, and the supernatant was removed and frozen on dry ice for transportation and subsequently stored at −20°C. Total length, body weight, and gonadal weight were determined for each fish. Gonads were removed and fixed in Bouin’s solution for 6–8 hr before storage in 70% IMS before processing for histologic analysis. Scales were collected from fish during the second adult trial to determine the age of individual fish.

In adult fish, we calculated condition factor (*K* ) for each individual using the formula *K* = [weight (g) × 100] ÷ (length)^3^. Gonadosomatic index (GSI) was calculated in these fish using the formula GSI = (gonad weight × 100) ÷ (total body weight – gonad weight).

### Measurement of VTG.

VTG was quantified in whole-body extracts in ELS fish and in plasma in adult fish using a carp-VTG enzyme-linked immunosorbant assay (ELISA) that has been validated for use in the roach (Tyler et al. 1999). For ELS fish, whole-body homogenates were prepared by defrosting the bodies of individual fish on ice and homogenizing with a phosphate-buffered saline, 0.05% Tween, and 1% bovine serum albumin, pH 7.4 (1 mL buffer/g of fish). After centrifugation at 15,000 rpm, the supernatant was removed and stored at −20°C until assayed in the VTG ELISA.

### Gonadal histology.

For ELS roach, tissue blocks for gonad sectioning were prepared by cutting the fish trunk either side of the dorsal fin. Samples were then embedded in paraffin wax, sectioned at 5 μm, mounted, and stained with hematoxylin and eosin. For adult roach, three sections were prepared from each gonad, one section from each of the anterior, middle, and posterior areas, providing a total of six gonad sections per fish. The sections were embedded in paraffin, sectioned at 5 μm, mounted, and stained with hematoxylin and eosin. All sections were analyzed by light microscopy.

### Statistical analyses.

All statistical analyses were carried out using Sigmastat (version 2.0; SPSS Inc. Chicago, IL, USA). Statistical significance was accepted at *p* < 0.05 for all comparisons. We assessed intergroup differences using one-way analysis of variance (parametric, for normalized data) or Kruskal-Wallis test (non-parametric). Multiple comparisons tests were performed, where appropriate, using post hoc analyses for parametric (Student-Newman-Keuls test) or nonparametric data (Dunn’s method). Histopathology data sets were analyzed for differences between treatment groups using chi-square analysis.

## Results

### Concentrations of steroid estrogens and alkyl-phenolic chemicals in the exposure effluents.

**Figure 2:**
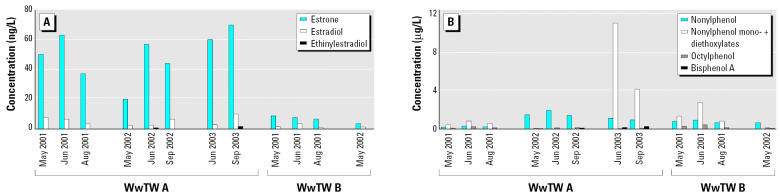
Measured concentrations of steroidal estrogens (*A*) and alkylphenolic chemicals (*B*) in full-strength (100%) effluent at both study sites during the experiments.

The concentrations of known estrogenic chemicals in the two effluents varied during the periods of study. In general, we observed that concentrations of steroid estrogens were higher in the effluent at WwTW A compared with that at WwTW B (Figure 2A). We detected the synthetic estrogen 17α-ethinylestradiol intermittently and only at WwTW A (up to 1.5 ng/L; limit of detection = 0.5 ng/L).

### Feminized responses in roach exposed to WwTW effluent during early life (exposed from fertilization to 300 dph).

#### Measured concentrations of treated wastewater effluent.

Measured concentrations of WwTW effluent in the mesocosm tanks remained close to nominal in all treatments. At WwTW A, measured concentrations of effluent during the roach exposures between fertilization and 60 dph were 0 ± 0% (tap water control), 0 ± 0% (river water control), 15.0 ± 1.3%, 24.9 ± 1.4%, 42.6 ± 2.2%, and 100 ± 0% (mean ± SEM). From 50 dph to 200 dph, the only remaining effluent treatment group was the full-strength effluent (100 ± 0%). At WwTW B, measured concentrations of treated effluent during the roach exposure between fertilization and 60 dph were 0 ± 0%, 8.0 ± 0.7%, 17.8 ± 0.9%, 36.6 ± 1.6%, 78.3 ± 1.0%, and 100 ± 0%. Between 50 dph and 100 dph, the measured exposure regimes were 0 ± 0%, 17.0 ± 1.0%, 33.6 ± 1.3%, 77.8 ± 0.8%, and 100 ± 0%. For the period between 100 dph and 200 dph, measured concentrations were 0 ± 0%, 12.4 ± 0.8%, 33.9 ± 1.3%, and 81.0 ± 2.5%, and for the period between 200 dph and 300 dph, 0 ± 0%, 15.7 ± 0.6%, 35.1 ± 0.6%, and 77.0 ± 0.4% (mean ± SEM). The temperature of the effluent/water fluctuated with the ambient temperature in all exposure tanks at both exposure WwTWs. There were no significant differences in water temperature between the various treatments at any one time point at either WwTWs (*p* > 0.05). There were differences in growth of the fish between treatments at each sampling point, but this was not related to the concentration of the effluents.

#### VTG induction.

**Figure 3:**
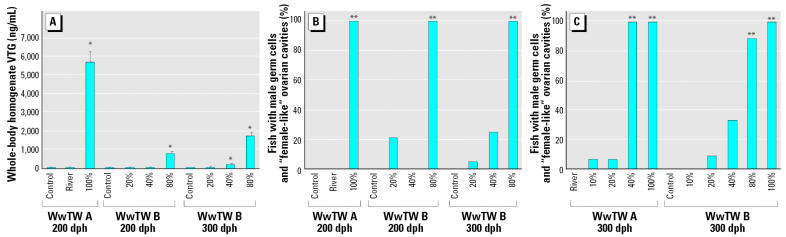
ELS study. (*A* ) Mean measured concentrations of VTG in whole-body homogenates of fish exposed from fertilization to 200 dph and 300 dph. (*B*) Percentage of fish with male germ cells and “female-like” reproductive duct exposed to WwTW effluents from fertilization to 200 dph and 300 dph. (*C*) Percentage of fish with male germ cells and “female-like” ovarian cavities after exposure to WwTW effluent from fertilization to 60 dph followed by depuration in clean water to 300 dph. *p < 0.05, and **p < 0.001 compared with controls.

Analysis of whole-body VTG at 200 dph (Figure 3A) showed that fish exposed to full-strength effluent at WwTW A had significantly higher titers of VTG (8-fold higher; 5,684 ± 546 ng/mL) compared with fish in the highest effluent concentration exposure group at WwTW B (80% effluent; 771 ± 122 ng/mL). This difference in plasma VTG in roach mirrored the differences in the effluent concentrations of steroid estrogens. At 300 dph, there was a concentration-dependent induction of VTG in roach at WwTW B, and in the 80% effluent a doubling in the body content of VTG (1709 ± 211 ng/mL), compared with fish at 200 dph. Fish that had been exposed to the various effluent concentrations for 60 days (to 60 dph) and then held in clean water to 300 dph contained little body VTG (between 29 ± 8 and 108 ± 42 ng/mL).

#### Gonadal development.

**Figure 4:**
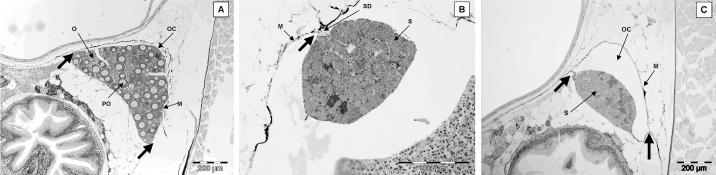
Gonadal histology of ELS roach after exposure to 100% effluent. (*A*) Control female. (*B*) Control male. (*C* ) Effluent-exposed male. Abbreviations: M, mesentery; O, oogonia; OC, ovarian cavity; PO, primary oocytes; S, spermatogonia; SD, sperm duct. Large arrows indicate points of attachment of the gonad to the mesentery.

Gonadal phenotypes of fish at both 200 dph and 300 dph included males and females and fish that had not completed sexual differentiation. The gonad of undifferentiated fish contained several primordial germ cells surrounded by stromal cells, and the gonad was attached to the body wall by a single point of attachment, the mesogonium. Definitive female fish (Figure 4A) contained ovaries with the gonad attached to the mesentery by two points of attachment forming the ovarian cavity (female reproductive duct). Two types of germ cells were observed in phenotypic female fish. In some individuals, ovaries contained only oogonia, but in other females at a more advanced stage of development and with larger ovaries, both oogonia and primary oocytes in the perinucleolar stage were present. There were no obvious differences in the development of ovaries in the controls compared with the effluent-exposed females. In presumptive males in controls (Figure 4B), the testes had a single point of attachment to the mesentery, forming the sperm duct. The testes of phenotypic males in the controls contained either spermatogonia A or both spermatogonia A and B. Several individuals had testes at a very advanced stage of development containing spermatogonia A and B, spermatocytes, and spermatids. Some male fish that had been exposed to either WwTW A or WwTW B effluent had feminized reproductive ducts. These testes contained male germ cells (spermatogonia A, and both spermatogonia A and B in more advanced fish) but were connected to the body wall by two distinct points of attachment forming a “female-like” duct or ovarian cavity (Figure 4C). There was no evidence of disruption of germ cell development in any of the fish examined in any of the treatments.

The frequency of ovarian cavity or “female-like” reproductive ducts in sexually differentiated fish containing male germ cells is shown in Figure 3B. At 200 dph in 80% (WwTW B) and 100% (WwTW A) effluent exposure groups, all fish with male germ cells had feminized reproductive ducts. At 300 dph (WwTW B), the presence of an ovarian cavity in fish with male germ cells was positively correlated with the concentration of effluent. The only statistically significant difference, however, was for the 80% effluent fish (compared with controls, *p* < 0.001). Many of the male fish exposed to WwTW effluent for 60 days and then maintained in clean water for a further 240 days contained an ovarian cavity. At WwTW A, all “males” exposed to 40% and 100% effluent during early life retained a feminized reproductive duct (Figure 3C). At this WwTW, low numbers of depurated males that had been exposed to 10% and 20% effluent during early life had an ovarian cavity at 300 dph. None of the male fish in the river water control at WwTW A had a feminized duct. At WwTW B, again none of the males in the controls, and in this case also none of the males in the depurated 10% effluent exposure group, contained an ovarian cavity.

### Feminized responses in adult postspawning roach exposed to WwTW effluents: adult exposure 1 (WwTW A only).

#### Measured concentrations of treated wastewater effluent.

Measured concentrations of WwTW effluent in the mesocosm tanks remained close to nominal in all treatments. The measured concentrations of treated effluent were 0 ± 0% (river water control), 26.5 ± 0.9%, 48.7 ± 1.0%, and 100 ± 0% (mean ± SEM).

#### Somatic and gonad growth.

**Figure 5:**
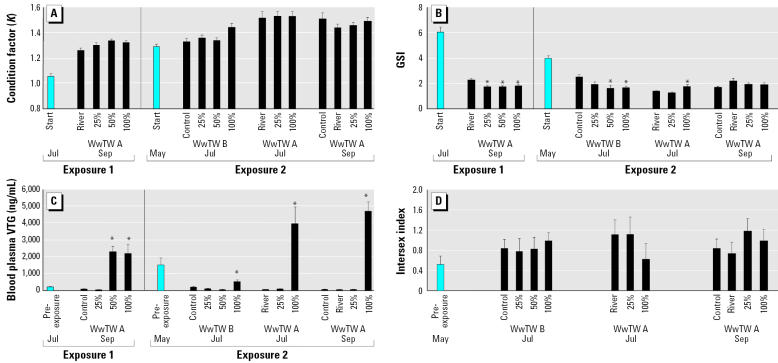
Pre/postspawning studies showing effects of exposure to WwTW effluents on (*A*) condition factor (*K* ) and (*B*) GSI. (*C* ) VTG concentrations in blood plasma in adult male roach. (*D*) Intersex index of adult fish in exposure 2. *p < 0.05 compared with control.

The condition factor of the fish increased during the trial across all the treatments. There was a significant difference in condition factor between control and 50% effluent-exposed fish only (enhanced condition in the 50% effluent fish, *p* < 0.05; Figure 5A). The GSI of the fish decreased during the trial across all the treatments in line with normal seasonal patterns for sexual development (Figure 4B). At the end of the trial, the GSI in all of the effluent exposure groups was significantly lower than the GSI in the males kept in river water (*p* < 0.05; Figure 5B).

#### Plasma VTG concentrations.

The concentration of plasma VTG in male fish before the effluent exposures in July was 226 ± 67 ng/mL; at the end of the trial in September in the river control fish, it was 91 ± 22 ng VTG/mL. Plasma VTG in the fish exposed to 25% effluent was 38 ± 15 ng/mL (no induction). In fish exposed to 50% and 100% effluent, the plasma VTG was significantly higher than in the controls, at 2,332 ± 329 ng/mL and 2,223 ± 561 ng/mL, respectively (*p* < 0.05; Figure 5C).

#### Gonad development.

**Figure 6:**
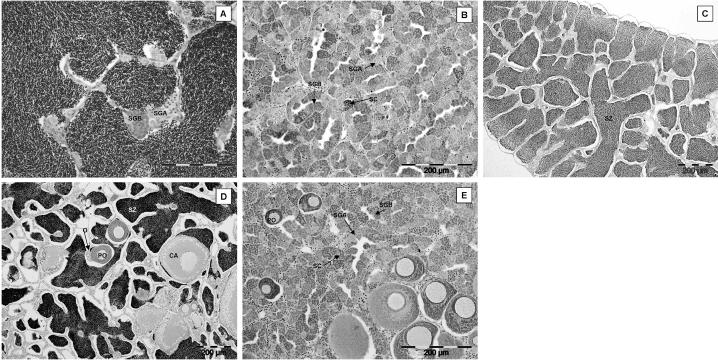
Pre/postspawning study (adult exposures 1 and 2) showing gonadal histopathology of male and intersex roach. (*A* and *B*) Gonads of males with no previous exposure to estrogen. In July (*A*), the testis was filled with spermatozoa (SZ), and cysts of spermatogonia A (SGA) and spermatogonia B (SGB) were also visible. In September (*B*), testes of male roach were normal, containing cysts of SGA, SGB, and spermatocytes (SC); there were no obvious differences between the testes of effluent-exposed fish and river water controls. (*C–E* ) Gonads of males with previous exposure to estrogen. In July, the testis was filled with SZ (*C* ), and some males were intersex (*D*). The testis contained SZ, oogonia (O), primary oocytes (PO), and larger oocytes in the cortical alveolus stage (CA). In September (*E* ), the gonads of these intersex fish contained cysts of SGA, SGB, and SC, together with PO.

All of the 12 spermiating male fish sampled in July had large amounts of spermatozoa contained in the testis lobules. A section through a typical testis of a 3+ years spermiating male roach is shown in Figure 6A. We identified a single intersex fish, and it contained a small number of primary oocytes in the perinucleolar stage surrounded by testicular tissue.

Spermiation had ceased in males sampled in September. These testes contained spermatogonia A, spermatogonia B, and spermatocytes but no spermatozoa (Figure 6B). One fish sampled from the control river water exposure group was identified as intersex and contained a small number of primary oocytes in the perinucleolar stage. There were no obvious differences between the testes of fish sampled from the effluent exposure groups and the river water controls.

### Adult exposure 2 (WwTWs A and B).

#### Measured concentrations of treated sewage effluent.

Measured concentrations of WwTW effluent in the mesocosm tanks remained close to nominal in all treatments.

At WwTW A, the measured concentrations of effluent between May, when the exposure started, and July were 0 ± 0% (tap water control), 0 ± 0% (river water control), 24.4 ± 0.9% (25% effluent), and 100 ± 0% (full-strength effluent; mean ± SEM). Between July and September, the measured concentrations of effluent were 0 ± 0% (tap water control), 0 ± 0% (river water control), 27.5 ± 1.7% (25% effluent), and 100 ± 0% (full-strength effluent; mean ± SEM). At WwTW B, measured concentrations of treated sewage effluent between May and July (when the exposure at this WwTW was terminated) were 0 ± 0% (tap water control), 25.1 ± 1.3% (25% effluent), 46.6 ± 1.5% (50% effluent), and 100 ± 0% (full-strength effluent; mean ± SEM).

#### Somatic and gonad growth.

The roach used in this trial were of mixed age, ranging between 3+ and 8+ years, with most in the 5+ year class. We found no clear correlates between age and any of the effects observed. The condition factor of the fish in the control group and all the treatment groups was higher at the termination of the experiments compared with the fish sampled at the start of the study (1.29 ± 0.02; Figure 5A). At WwTW A in both July and September, there were no significant differences in condition factor between control river-water–exposed and effluent-exposed fish. At WwTW B, the only significant difference in condition factor occurred between the controls and 100% effluent-exposed fish (an enhanced condition in the effluent-exposed fish; *p* < 0.05). As in adult exposure 1, the GSI decreased during the experiment across all the treatments in line with normal seasonal patterns for sexual development (Figure 5B). At WwTW A in July, the GSI in the 100% effluent-exposed fish was higher than in the river-water–exposed controls (*p* < 0.05), but there were no differences in GSI between the effluent-exposed fish and the control fish sampled in September. At WwTW B, the GSI was lower in the 50% and 100% effluent-exposed fish compared with the controls, indicating suppression in gonadal recovery after spermiation.

#### Plasma VTG concentrations.

The plasma VTG concentrations in fish at the start of the study were elevated (1,531 ± 426 ng/mL), indicating a previous exposure to an estrogenic stimulus (Figure 5C). At WwTW A in July, 2 months after the start of the experiment, males in the control river water and in the 25% effluent had plasma VTG concentrations of 62 ± 9 ng/mL and 94 ± 27 ng/mL, respectively, showing a clearance of VTG from the circulation. Fish exposed to 100% effluent had an elevated concentration of plasma VTG (~ 3-fold above the preexposure concentration, at 4,017 ± 1,013 ng/mL; *p* < 0.05). A similar pattern of effect occurred for these fish in September. At WwTW B in July, the plasma VTG concentration in male fish in the 100% effluent was 525 ± 136 ng/mL (significantly higher than in the controls; *p* < 0.05).

#### Gonad development.

We found intersex fish in both control and effluent treatment groups in exposure 2. The degree of disruption in the testes was classified into one of three categories: 0 = normal (no oocytes), 1 = a few oocytes (≤10 per section and principally composed of primary oocytes), and 2 = many oocytes (> 10 oocytes per testes section, many of which were often in the cortical alveolus stage of development). We found no differences between treatments and sampling points, and numbers and severity of the intersex condition (Figure 5D; *p* > 0.05). Furthermore, there was no effect of age on severity of the intersex condition (*p* > 0.05).

All fish sampled in May were producing sperm. All testis sections contained spermatozoa within the lobules and dispersed cysts of spermatogonia A and spermatogonia B. A section through a typical testis of a normal spermiating male roach for this study group is shown in Figure 6C. All intersex fish also had spermatozoa contained within lobules (a typical testis of an intersex roach is shown in Figure 6D). In fish sampled in July and September, both normal males and intersex fish were found in every effluent exposure group and in the controls. Testes in normal males were histologically very similar to those analyzed in adult exposure 1 (Figure 6B). The intersex condition included clusters of primary oocytes nested in an otherwise normal testicular tissue and, in the more severe condition, contained secondary oocytes (Figure 6E).

## Discussion

The concentrations of both steroid estrogens and alkylphenolic chemicals in the two study effluents were comparable with those measured in other WwTW effluents in the United Kingdom and worldwide (Lee and Peart 1998; Spengler et al. 2001; Ternes et al. 1999). Differences in concentrations of the estrogenic chemicals measured in the two study effluents reflect either differences in the influent, differences in effluent dilution, and/or the different treatment processes employed in the works, as established in other studies (Kirk et al. 2002; Svenson et al. 2003; Ternes et al. 1999). The effluent content of both steroid estrogens and alkylphenolic chemicals was also variable within each of the works studied, even for samples collected as 7-day composites and even over the relatively short interval of only 2 months. At WwTW A, for example, the estrone content varied by more than 3-fold, and the concentration of nonylphenol mono-and diethoxylates varied by more than 9-fold. Seasonal differences may, at least in part, be due to changes in ambient temperature, which can affect microbiological populations and therefore the efficacy of biological treatments and/or high rainfall, which can result in dilution of influent to the WwTW and a reduced retention time through the works. This finding emphasizes the need for caution when single-point measures are used (as they often are) to assess steroidal estrogen and alkylphenolic chemical contents in WwTW effluents.

Both effluents induced a vitellogenic response in both juveniles and adults; however, the responses in fish at WwTW A were greater than those in fish at WwTW B, mirroring the higher concentrations of steroid estrogens at WwTW A. In the ELS exposure study, the vitellogenic response to full-strength (100%) effluent at WwTW A (at 200 dph) was an order of magnitude higher than the response to 80% effluent at WwTW B. Further exposure of the juvenile roach in the 80% effluent at WwTW B to 300 dph resulted in a further 2-fold increase in the VTG titer. This increase in VTG may have been as a consequence of a higher content of estrogenic chemicals during the 200–300 dph exposure period; however, no analytical chemistry was carried out at this time to confirm this. Alternatively, the increased titer of VTG in these fish may have resulted from the bio-concentration of the estrogenic chemicals in the fish from the effluent, which is known to occur for both steroid estrogens and alkylphe-nolic chemicals (Larsson et al. 1999). In the adult exposures, for males derived from the wild and exposed to full-strength effluent at WwTW B, the concentration of plasma VTG was lower (~ 33%) than in the preexposure fish, suggesting that the environment from which these fish were derived was more strongly estrogenic than was the effluent at WwTW B. The transient nature of the VTG induction was demonstrated in the ELS fish. At the end of the depuration study, there was a subsequent clearance of VTG (to concentrations not significantly different from controls) in fish previously exposed to effluent.

In the ELS exposure, there were differences in growth of fish between the treatments. Differences in growth rate may affect the timing of sexual differentiation and affect sex ratios (reviewed by Devlin and Nagahama 2002). However, at the end of the trial (300 dph), not all fish had completed sexual differentiation, and therefore we were not able to determine any differences in sex ratios between the treatments.

Adult fish across all treatment groups had an increased condition factor in both of the postspawning studies. The increased condition of the fish with time across the treatments was expected because energy directed to fuel rapid gonad growth during recrudescence is directed to support somatic growth in postspawning fish. The higher concentrations of effluent appeared to enhance condition recovery, and this may have occurred as a consequence of a greater availability of natural food, or because less energy was partitioned to gonad recovery (an associated inhibitory effect on testis recrudescence). Other studies have demonstrated that exposure to estrogenic chemicals, particularly alkylphenolic compounds commonly found in WwTW effluent, has a negative effect on testicular growth (Cardinali et al. 2004; Jobling et al. 1996; Magliulo et al. 2002). The reduced gonad mass (GSI) in the adult postspawning trials was in line with normal seasonal patterns for sexual development (the fish ceased producing sperm). There was no consistent pattern in the effect of effluent exposure on gonad recrudescence postspawning. In some cases, there appeared to be an inhibitory effect however, in others, a positive effect was observed, which may be due to an increased condition factor of these fish.

Exposure of juvenile roach to high concentrations of treated WwTW effluent from fertilization to 200 dph and 300 dph resulted in the feminization of the male reproductive duct. At 200 dph, all males in the higher effluent concentrations at both WwTWs had feminized ducts. At WwTW B, there was no concentration-related effect on duct feminization at 200 dph, but there was at 300 dph, when more fish had completed sexual differentiation (and thus their gonadal sex could be determined). Fish exposed to effluent at both WwTWs during early life and then transferred to clean water at 60 dph to depurate had disrupted ducts at the end of the study, indicating this effect was permanent. This supports previous studies that have shown feminized ducts induced by estrogen and estrogenic mixtures persist (Gimeno et al. 1997; Rodgers-Gray et al. 2001; van Aerle et al. 2002). The data presented here show that the window for disruption of duct development in roach includes the period between fertilization and 60 dph, before gonadal sexual differentiation is visible histologically. Feminization of the reproductive duct in fish has been induced in male fish in laboratory exposure to various estrogenic chemicals, including estradiol (Brion et al. 2004; Gimeno et al. 1996), ethinylestradiol (van Aerle et al. 2002), and the alkylphenolic compound 4-*tert*-pentylphenol (Gimeno et al. 1997, 1998), but only at relatively high exposure concentrations. The concentrations of steroid estrogens and alkylphenolic chemicals in WwTW B effluent were considerably lower than for those found to induce feminization of reproductive ducts in laboratory studies (Gimeno et al. 1996, 1998; van Aerle et al. 2002). The functional significance of duct feminization in males has been shown to depend on the severity of the condition, and feminized roach with severely disrupted ducts have a decreased fertility (Jobling et al. 2002b).

The field evidence that WwTW effluents induce germ cell disruption in wild roach is substantial (Jobling et al. 1998). The lack of germ cell disruption in any of the fish examined in the ELS study or in effluent-exposed adult males derived from estrogen-free environments indicated that either the estrogenic potency of effluents tested was not sufficient to induce this feature of feminization, or that germ cell disruption is a consequence of a longer term exposure to treated wastewater effluent. The longevity of exposure hypothesis is strongly supported by our recent findings that the severity of intersex in wild roach is age related (our unpublished data). In fish that had received exposure to estrogen before the exposure to the WwTW effluents, there was an apparent increase in the severity of the intersex condition, for both treatments and controls. Thus, this effect was not a function of the constituents in the WwTW effluents, but was possibly the consequence of a previously determined programming of germ cell disruption. The available data on intersex in roach thus suggest that oocytes in the testis of male roach arise either as a consequence of long-term exposure to estrogenic chemicals or as a consequence of programming during early life that manifests later in development as fish undergo sexual maturation.
